# Chronic Obstructive Pulmonary Disease-Related Non-Small-Cell Lung Cancer Exhibits a Low Prevalence of EGFR and ALK Driver Mutations

**DOI:** 10.1371/journal.pone.0142306

**Published:** 2015-11-10

**Authors:** Jeong Uk Lim, Chang Dong Yeo, Chin Kook Rhee, Yong Hyun Kim, Chan Kwon Park, Ju Sang Kim, Jin Woo Kim, Sang Haak Lee, Seung Joon Kim, Hyoung Kyu Yoon, Tae-Jung Kim, Kyo Young Lee

**Affiliations:** 1 Division of Pulmonology, Department of Internal Medicine, College of Medicine, The Catholic University of Korea, Seoul, Republic of Korea; 2 Department of Hospital Pathology, College of Medicine, The Catholic University of Korea, Seoul, Republic of Korea; Istituto dei tumori Fondazione Pascale, ITALY

## Abstract

Lung cancer and chronic obstructive pulmonary disease (COPD) are two major lung diseases. Epidermal growth factor receptor (EGFR) mutations, v‐Ki‐ras2 Kirsten rat sarcoma (KRAS) mutations and anaplastic lymphoma kinase (ALK) gene rearrangements represent driver mutations that are frequently assessed on initial evaluation of non-small-cell lung cancer (NSCLC). The present study focused on the expression of driver mutations in NSCLC patients presenting with COPD and further evaluated the association between NSCLC and COPD. Data from 501 consecutive patients with histologically proven recurrent or metastatic NSCLC were analyzed retrospectively. The patients underwent spirometry and genotyping of EGFR, ALK, and KRAS in tissue samples. Patient characteristics and expression of driver mutations were compared between the COPD and non-COPD groups.

Among 350 patients with spirometric results, 106 (30.3%) were diagnosed with COPD, 108 (30.9%) had EGFR mutations, 31 (8.9%) had KRAS mutations, and 34 (9.7%) showed ALK rearrangements. COPD was independently associated with lower prevalences of EGFR mutations (95% confidence interval [CI], 0.254–0.931, *p* = 0.029) and ALK rearrangements (95% CI, 0.065–0.600, *p* = 0.004). The proportions of EGFR mutations and ALK rearrangements decreased as the severity of airflow obstruction increased (*p* = 0.001). In never smokers, the prevalence of EGFR mutations was significantly lower in the COPD group than in the non-COPD group (12.7% vs. 49.0%, *p* = 0.002). COPD-related NSCLC patients exhibited low prevalences of EGFR mutations and ALK rearrangements compared with the non-COPD group. Further studies are required regarding the molecular mechanisms underlying lung cancer associated with COPD.

## Introduction

Lung cancer and chronic obstructive pulmonary disease (COPD) are two important lung diseases associated with smoking[1]. Lung cancer occurs roughly fivefold more frequently in COPD patients compared with non-COPD patients, and the presence of COPD is associated with increased mortality in lung cancer patients [2]. Moreover, 50–70% of lung cancer patients show spirometric evidence of COPD [3–5]. Tobacco smoking is widely accepted as a common pathogenic cause of COPD and lung cancer [6]. However, even after considering the effects of smoking, the presence of COPD is an independent risk factor for lung cancer development [7]. Furthermore, lung cancer in COPD patients has been associated with worsened regional severity of emphysema [8,9]. Even with strong epidemiological associations reported in literature, the mechanisms of the link between COPD and lung cancer are not clearly explained.[10]

The association between COPD and lung cancer can also be evaluated from a molecular perspective. Driver mutations—including epidermal growth factor receptor (EGFR) mutations and anaplastic lymphoma kinase (ALK) rearrangements—have clinical significance in the treatment of non-small-cell lung cancer (NSCLC). Sensitivity to EGFR tyrosine kinase inhibitors (TKI) depends on the expression of EGFR mutations [11–14]. Mutation of the EGFR signaling pathway has been implicated in the development of NSCLC [15,16]; indeed, several studies have classified NSCLC into two distinct categories: EGFR-mutated and EGFR-wild type NSCLC [12,14]. For the treatment of EGFR-mutant lung cancer, EGFR TKIs are recommended as first-line regimens because of their superior efficacy to platinum doublet regimens in terms of progression-free survival [13]. The roles of EGFR mutations are important because of their predictive abilities in targeted therapy; however, other driver mutations are also important in NSCLC [17]. ALK rearrangements have been newly discovered in NSCLC [18], and crizotinib, a multi-targeted TKI, was shown to be effective in NSCLC harboring ALK rearrangements [19]. For advanced-stage lung adenocarcinoma, testing for EGFR mutations and ALK rearrangements are routinely recommended to select patients for targeted therapy [20]. Additionally, v‐Ki‐ras2 Kirsten rat sarcoma (KRAS) mutations occur in approximately 25% of NSCLCs, and preclinical and clinical studies of novel therapies targeting KRAS downstream pathways are under progress [21]. EGFR and KRAS mutations and ALK rearrangements are major driver mutations to be considered in the initial evaluation of NSCLC.

Previous studies suggest that the pathogenesis of COPD is closely associated with lung carcinogenesis, but few have reported the expression of driver mutations and the clinical characteristics of NSCLC patients with COPD. *Suzuki* et al. reported that the incidence of EGFR mutations was lower in NSCLC patients with COPD than in those without COPD [1].

Schiavon et al compared the molecular features of COPD-associated adenocarcinoma patients to patients with smoke-related adenocarcinoma without COPD. No differences were found between the two groups concerning EGFR mutation, while KRAS mutation was higher in smokers compared to COPD patients. [10] To date, large-sized studies investigating differences in the expression of driver mutations between COPD and non-COPD lung cancer patients are lacking.

The aim of the present study was to evaluate the expression of the major driver mutations EGFR and KRAS mutations and ALK rearrangements in NSCLC patients with COPD to determine the association between the presence of COPD and driver mutations.

## Patients and Methods

### Study population

Data from 501 consecutive patients with histologically proven recurrent or metastatic NSCLC, who were admitted to Seoul St. Mary’s Hospital, Yeouido St. Mary’s Hospital, Incheon St. Mary’s Hospital and Bucheon St. Mary's Hospital at The Catholic University of Korea between January 2011 and April 2013, were analyzed retrospectively. All patients signed clinical consent forms. The patients underwent non-sequential, simultaneous panel genotyping of EGFR, ALK, and KRAS. The patients’ clinicopathological characteristics detailed in medical records were reviewed, including age, gender, smoking history, tumor histological type, initial tumor stage, EGFR and KRAS mutations, ALK rearrangements, and presence of COPD. NSCLC tumor pathology was classified according to the World Health Organization classification. Clinical staging of lung cancer was determined according to tumor, node, metastasis (TNM) staging using the standards of the Union for International Cancer Control (UICC), seventh edition [22]. Smoking status was categorized as “never” if <100 cigarettes were consumed during a lifetime and “ever” if otherwise. This study was approved by the Institutional Review Board of each participating hospital.

### Definition of COPD

Spirometry screening assessment was performed upon admission of the patients. COPD was defined as a predicted forced expiratory volume in 1 second (FEV1) /forced vital capacity (FVC) value ≤ 70% in accordance with the current Global Initiative of Chronic Obstructive Lung Disease (GOLD) guidelines [23]. Patients whose FEV1/FVC value was lower than 70% were assigned to the COPD group. Spirometric values were presented as the postbronchodilator results [23]. The severity of airflow obstruction in COPD was determined using the GOLD grading system: grade 1 (%FEV1 >80%), grade 2 (%FEV1 50–80%), grade 3 (%FEV1 30–50%), and grade 4 (%FEV1 <30%). One hundred and fifty-one patients without pulmonary function test results due to a poor general condition and an inability to undergo the studies were excluded.

### EGFR and KRAS mutation testing

EGFR mutations were defined as exon 19 deletion or exon 21 point mutations. We excluded patients with other more uncommon EGFR mutation profiles. Genotyping of EGFR and KRAS was performed by peptide nucleic acid (PNA)-mediated PCR clamping methods, such as the PNAClamp^TM^ EGFR MutationDetection Kit and PNAClamp^TM^ KRAS Mutation Detection Kit (PANAGENE, Inc., Daejeon, Korea), using real-time PCR [24]. PCR was performed in a total reaction volume of 20 μl including the template DNA, primer and PNA probe sets, and SYBR Green PCR master mix. The PCR control lacked the PNA probe and contained the wild-type template. The CFX96 PCR detection system was used to perform PCR.

### ALK fluorescent in situ hybridization (FISH)

**Fig 1:**
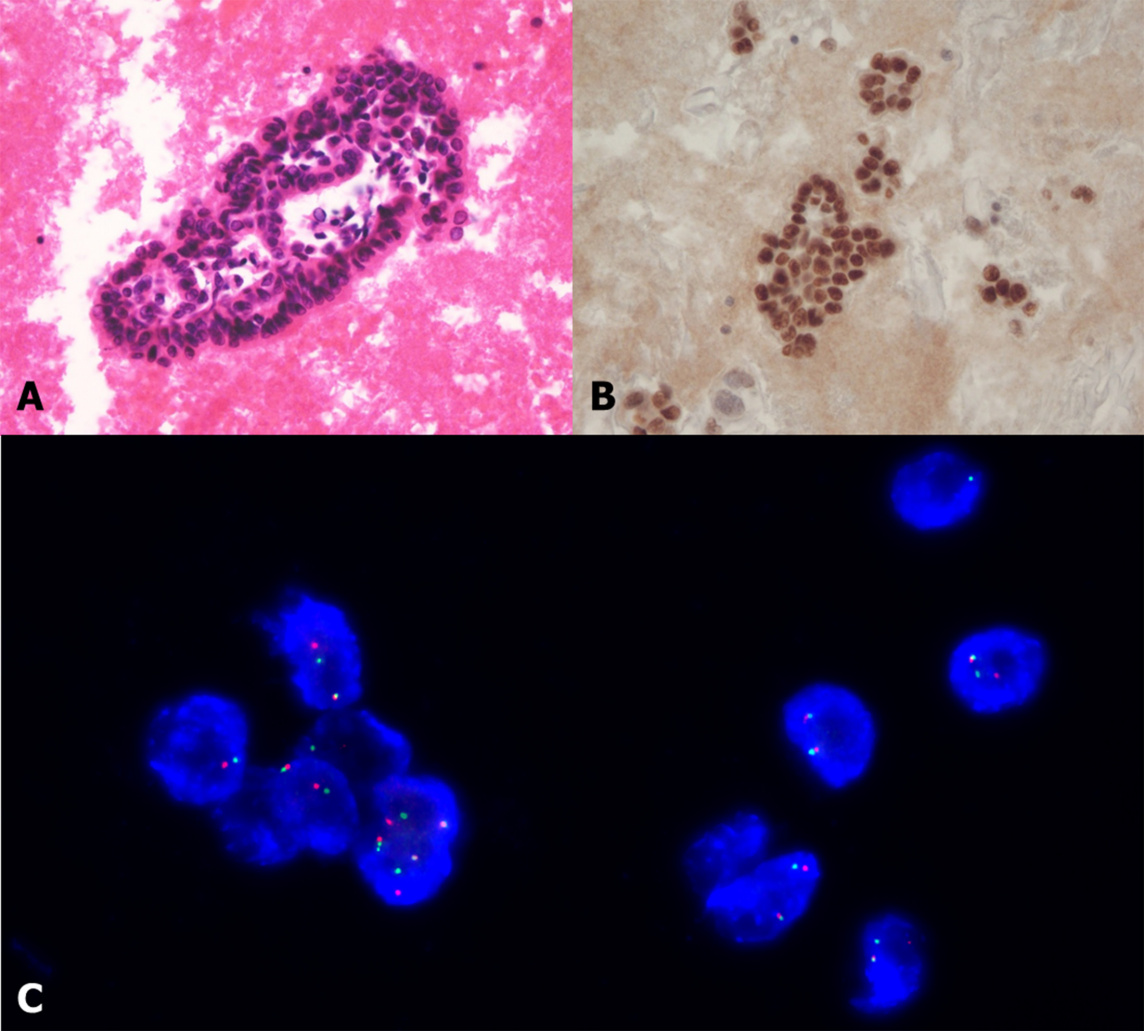
Representative photographs of a patient with ALK rearranged adenocarcinoma in pleural fluid cell blocks. (A) Tumor with H&E stain. (B) Tumor with positive TTF-1 stain. (C) FISH analysis interpreted as positive; 15 ALK rearranged cells in 57 tumor cells (ALK FISH split >15%). *Abbreviations* H&E: hematoxylin and eosin; TTF-1; thyroid transcription factor 1.

Specimens for FISH obtained from four hospitals were prepared simultaneously using a molecular analysis platform and were analyzed over a 3-day period at Yeouido St. Mary’s Hospital Central Molecular Laboratory [25]. FISH was performed on formalin-fixed paraffin-embedded (FFPE) tumor tissues using a break-apart probe specific to the ALK locus, the Vysis LSI ALK Dual Color Break Apart Probe (Abbott Molecular, Abbott Park, IL, USA). ALK rearrangement positivity was defined as a split signal or isolated red signal. A minimum two-probe diameter distance was required for determination of true positive signal splitting. Positive cases were defined as those with >15% of counted nuclei within tumor cells exhibiting a split signal or isolated red signal (Fig 1). To minimize technical bias, we used a specimen-specific assessment approach. In surgical resection specimens, 100 tumor cells were scored. An ALK FISH split signal rate <15% was interpreted as negative and that ≥15% as positive.

### Statistical analysis

Data are expressed as frequencies with percentages or as means with standard deviations. The basic characteristics of the study population were compared using the unpaired Student’s *t*-test for continuous variables and the chi-squared test for categorical variables. We used logistic regression analysis to examine the relationship between driver mutations and other clinical variables. Odds ratios and their 95% confidence intervals (CI) were computed. Goodness of fit was computed to assess the relevance of the logistic regression model. A linear-by-linear association test was employed to explore the associations between a decline in FEV1 and the expression of driver mutations. All statistical analyses were performed using SPSS software (ver. 15.0.0 for Windows; SPSS Inc., Chicago, IL, USA), and *p* < 0.05 was taken to reflect significance.

## Results

### Overall patient characteristics

After exclusion of 151 patients without pulmonary function test results, 350 patients with NSCLC were enrolled. Of these, 106 patients were selected for the COPD group and 244 for the non-COPD group. The mean age of the patients was 65.1 years; 192 (54.9%) were male, 199 (54.9%) were never smokers, and 17 (4.9%) had squamous cell carcinomas. The clinical stages of 226 patients (64.6%) were III or IV at the time of diagnosis. EGFR mutations were present in 108 patients (30.9%), KRAS mutations in 31 (8.9%) and ALK rearrangements in 34 (9.7%). The mean %FEV1 predicted value was 90.9%, and the mean FEV1 absolute value in liters was 2.32 (Table 1).

**Table 1:** Characteristics of 350 NSCLC patients and a comparison of the COPD versus non-COPD groups.

Characteristic	Overall	COPD	Non-COPD	*p-value*
	(n = 350)	(n = 106)	(n = 244)	
Age (mean)	65.08±10.68	69.78±9.49	63.03±10.54	*0*.*001*
Sex				
Male	192 (54.9%)	83 (78.3%)	109 (44.7%)	*0*.*001*
Female	158 (45.1%)	23 (21.7%)	135 (55.3%)	
Smoking History				
Never smoker^a^	199 (56.9%)	43 (40.6%)	156 (63.9%)	*0*.*001*
Ever smoker^a^	151 (43.1%)	63 (59.4%)	88 (36.1%)	
Former smoker	18 (5.1%)	6 (5.7%)	12 (4.9%)	
Current smoker	133 (38%)	57 (53.7%)	76 (31.2%)	
Histology				
Adenocarcinoma	318 (90.9%)	91 (85.8%)	227 (93%)	*0*.*029* ^*b*^
Non-adenocarcinoma	32 (9.1%)	15 (14.2%)	17 (7%)	
Squamous carcinoma	17 (4.9%)	9 (8.5%)	8 (3.3%)	
Adenosquamous carcinoma	6 (1.7%)	2 (1.9%)	4 (1.6%)	
NOS^d^	9 (2.6%)	4 (3.8%)	5 (2%)	
Clinical stage				
I	87 (24.9%)	25 (23.6%)	62 (25.4%)	*0*.*808* ^*c*^
II	37 (10.6%)	13 (12.3%)	24 (9.8%)	
III	80 (22.9%)	30 (28.3%)	50 (20.5%)	
IV	146 (41.7%)	38 (35.8%)	108 (44.3%)	
History of pulmonary tuberculosis	33 (9.4%)	11 (10.4%)	22 (9%)	*0*.*693*
Mean %FEV1 predicted value	90.85±22.72	77.61±21.2	96.6±20.91	*0*.*001*
Mean FEV1 absolute value (liters)	2.32±4.007	1.81±0.623	2.56±4.767	*0*.*001*

NSCLC, non-small-cell lung cancer; EGFR, epidermal growth factor receptor; KRAS, v‐Ki‐ras2 Kirsten rat sarcoma viral oncogene homolog; ALK, anaplastic lymphoma kinase; FEV1, forced expiratory volume in 1 second; COPD, chronic obstructive pulmonary disease

^a^ Smoking status was categorized as “never” if <100 cigarettes were consumed in a lifetime and as “ever” otherwise.

^b^Adenocarcinoma vs. all others

^c^Stages I and II vs. III and IV

^d^NOS: Not otherwise specified

### Patient characteristics and differences between the COPD and non-COPD groups: univariate analysis

**Fig 2:**
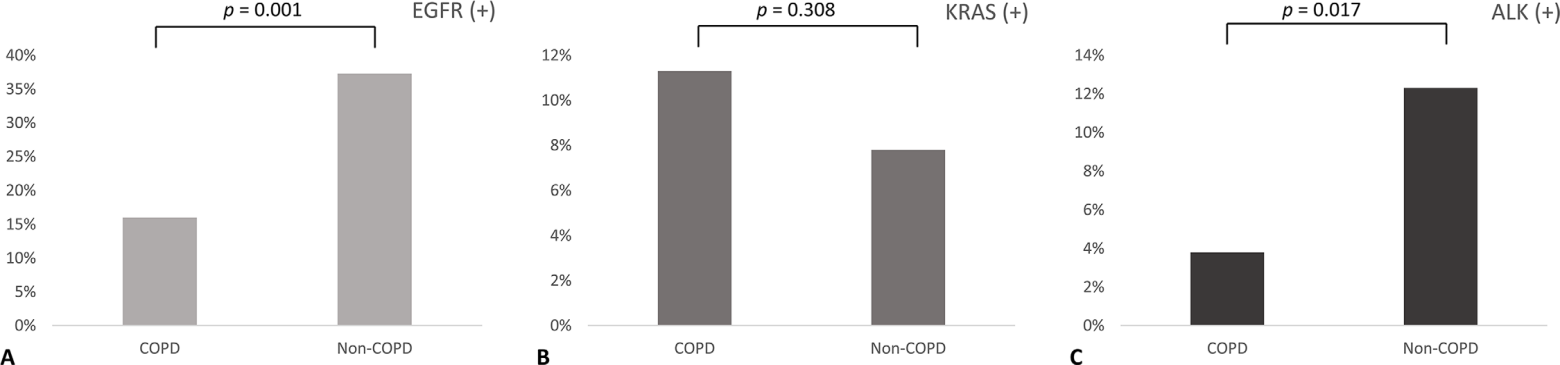
Comparison of prevalence of major driver mutations between COPD and non-COPD groups. (A) EGFR mutations (B) KRAS mutations and (C) ALK rearrangements.

In the COPD group, the mean age was higher (69.8 vs. 63.0 years, *p* = 0.001), and male patients were more common (78.3% vs. 44.7%, *p* = 0.001). Smokers were more prevalent in the COPD group (59.4% vs. 36.1%, *p* = 0.001), while adenocarcinomas were more prevalent in the non-COPD group (93% vs. 85.8%, *p* = 0.029). No significant differences in clinical stage were found between the two groups (Table 1). The proportion of overall patients expressing EGFR mutations was 30.9%, and these mutations were more prevalent in the non-COPD (37.3%) compared with the COPD group (16%) (*p* = 0.001). ALK rearrangements were significantly less prevalent in the COPD group (3.8% vs. 12.3%) (*p* = 0.013). As to KRAS mutation, no significant difference was found between two groups (Fig 2).

### Multivariate analysis of risk factors associated with EGFR mutations and ALK rearrangements

We performed multivariate analyses to identify the risk factors associated with EGFR mutations and ALK rearrangements. The variables found to be significant in the univariate analyses were introduced into a logistic regression model. The effects of age, gender, smoking, and histology were assessed by multivariate analyses. EGFR mutations were more common in females, never smokers, and patients with adenocarcinomas (Table 2). COPD was independently associated with lower prevalences of EGFR mutations (95% CI, 0.254–0.931, *p* = 0.029) and ALK rearrangements (95% CI, 0.065–0.600, *p* = 0.004). The odds ratio was 0.487 for EGFR mutations (95% CI, 0.254–0.931, *p* = 0.029) and 0.197 for ALK rearrangements (95% CI, 0.065–0.600, *p* = 0.004).

**Table 2:** Multivariate analysis of patient characteristics associated with EGFR and ALK mutation statuses.

	EGFR			ALK		
	Odds Ratio	95% CI	*p*-value	Odds Ratio	95% CI	*p*-value
COPD	0.197	0.065–0.600	0.004	0.487	0.254–0.931	0.029
Age	0.992	0.959–1.025	0.616	1.005	0.981–1.030	0.678
Male	4.217	1.478–12.030	0.007	0.453	0.236–0.868	0.017
Smoking	1.188	0.470–3.004	0.716	0.468	0.234–0.937	0.032
Adenocarcinoma	3.113	0.686–14.117	0.141	3.662	1.062–12.624	0.040

COPD, chronic obstructive pulmonary disease; EGFR, epidermal growth factor receptor; ALK, anaplastic lymphoma kinase.

### GOLD stages

**Fig 3:**
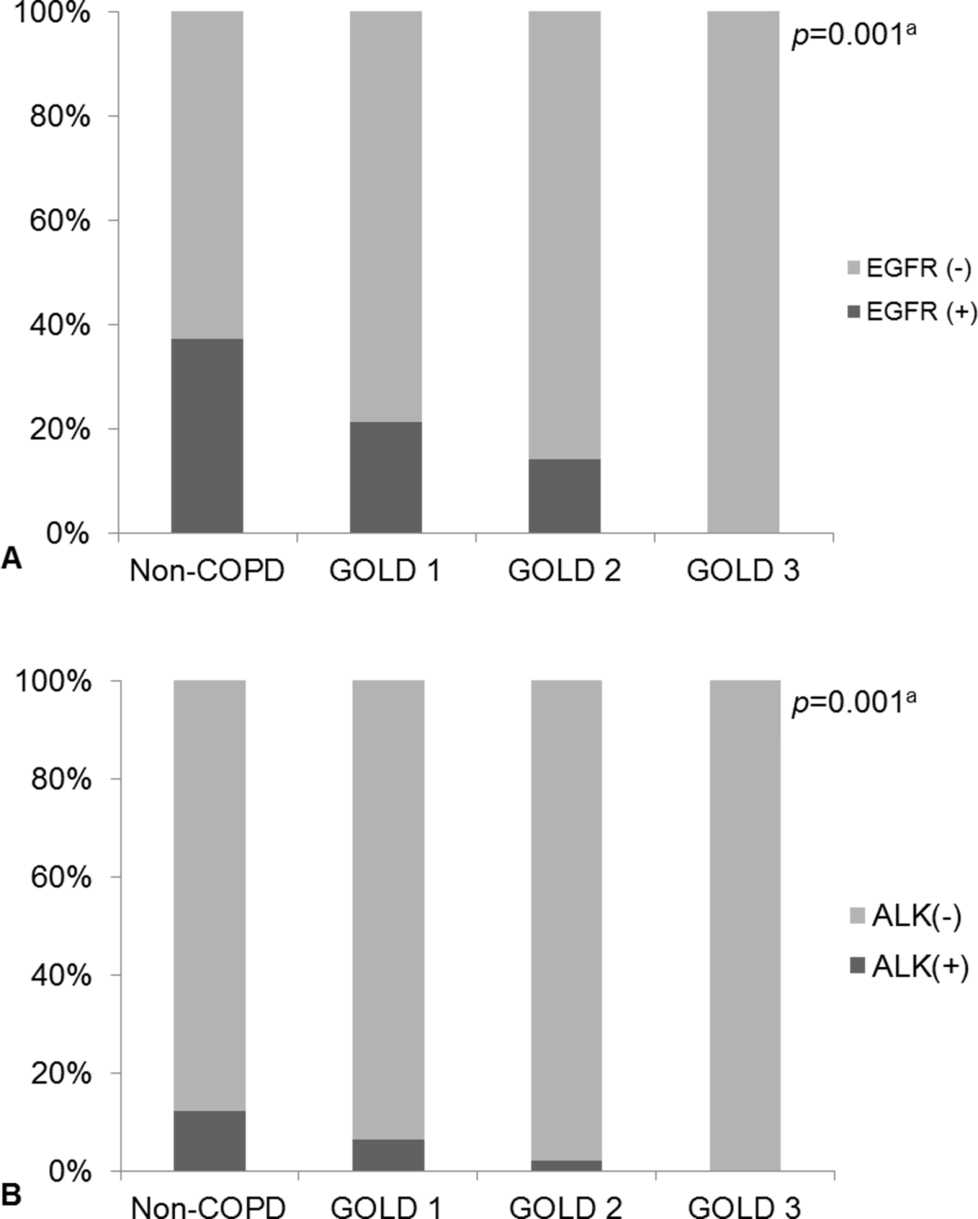
The proportion of patients with EGFR mutations or ALK rearrangements decreased with the severity of airflow obstruction, as assessed by the GOLD stage. (A) EGFR mutations and (B) ALK rearrangements. ^a^ *p*-values were calculated by linear-by-linear association test.

Among the 106 COPD patients, 47, 48, 11, and 0 were classified as GOLD stages 1, 2, 3, and 4, respectively. To explore whether or not the severity of airflow obstruction is significantly correlated with the prevalence of gene mutations, we divided the patients of the non-COPD group into GOLD stages 1, 2, and 3. The proportion of those with EGFR mutations was inversely proportional to the severity of airflow obstruction (*p* = 0.001). Furthermore, the presence of ALK rearrangements also decreased as airflow obstruction increased (Fig 3).

### Differences in driver mutations between the COPD and non-COPD groups among never smokers

**Fig 4:**
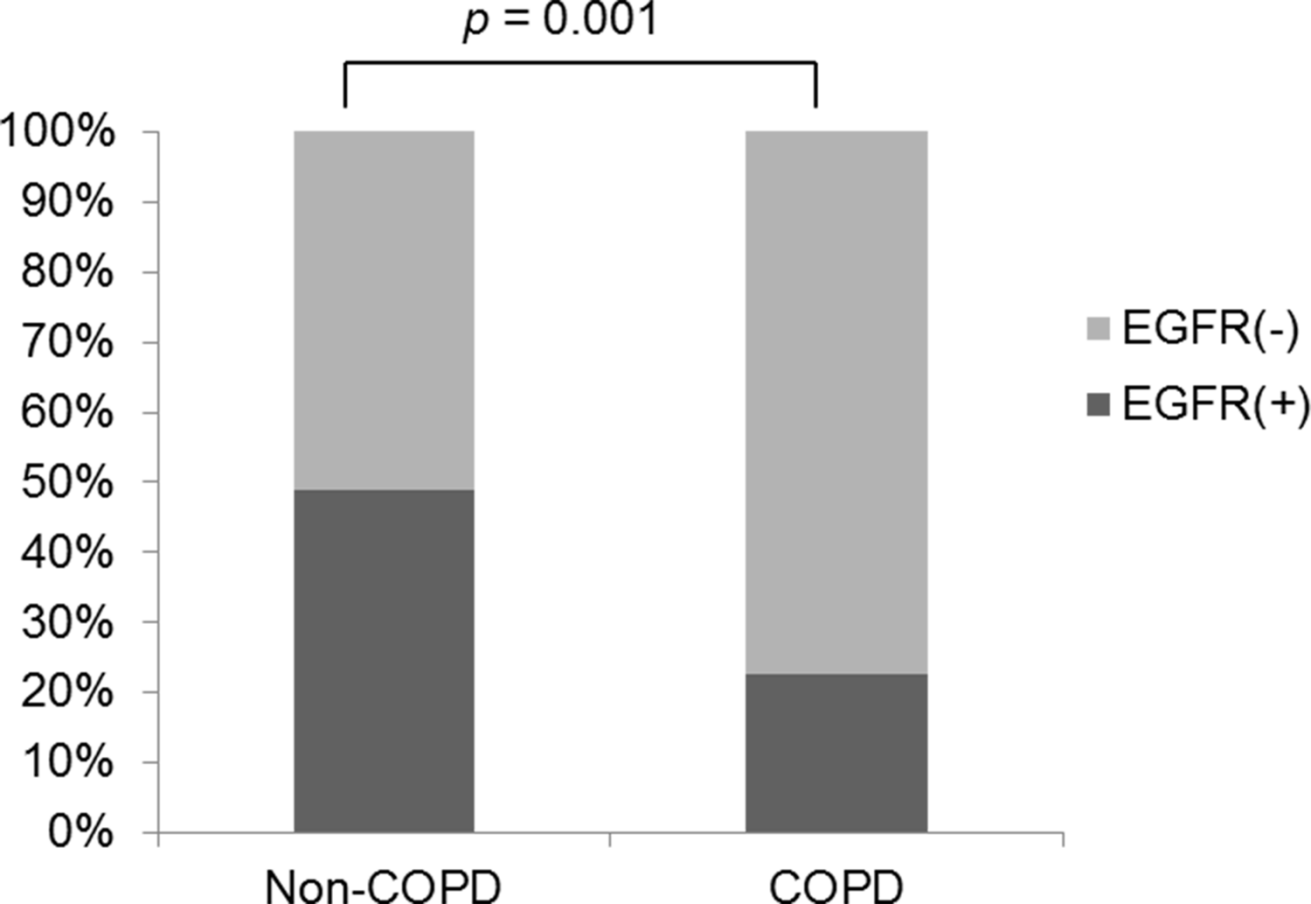
Association between EGFR mutations and the presence of COPD in non-smokers (n = 199).

Excluding the effect of smoking, we determined the association between COPD and EGFR mutations in 199 never-smoker NSCLC patients. The proportion of patients with EGFR mutations was significantly lower in the COPD group than the non-COPD group (12.7% vs. 49.0%, *p* = 0.002). No statistically significant difference in the prevalence of ALK rearrangements was found between the two groups (Fig 4).

## Discussion

The aim of our study was to evaluate the prevalence of driver mutations in patients with NSCLC and COPD according to the severity of airway obstruction. Patients with COPD-related NSCLC had low prevalences of EGFR mutations and ALK rearrangements, which were proportional to the decline in FEV1. Furthermore, COPD was also significantly correlated with low prevalences of EGFR mutations and ALK rearrangements in non-smoker NSCLC patients. However no significant difference between the COPD and non-COPD group in expression of KRAS mutation was found.

Our study results are consistent with previous findings that EGFR mutations were less prevalent in males and smokers and more prevalent in adenocarcinomas [26–29]. However, there are few data clarifying the correlation between COPD and major driver mutations in NSCLC patients. A recent study by *Hashimoto* et al. showed that EGFR mutations were more prevalent in a non-COPD group compared with a COPD group [30]. A previous study showed that the expression of EGFR mutations was proportionally correlated with a decline in FEV1; however, the association was not valid in multivariate analysis [1]. Our study demonstrated that COPD was independently associated with a lower prevalence of EGFR mutations among NSCLC patients, after taking age, gender, smoking, and histology into account, and the number of subjects enrolled was larger. In addition, our study showed that the prevalence of ALK rearrangements in NSCLC was lower in the COPD than the non-COPD group.

Airflow obstruction severity defined by %FEV1 was proportional to the prevalences of EGFR mutations and ALK rearrangements in our study. Smoking is a possible reason for the correlation. Smoking also induces bronchial epithelial changes in COPD patients [31]. Adenocarcinoma development may involve different pathways between smokers and never smokers. In smokers, smoking-related carcinogens favor KRAS mutations; on the other hand, EGFR mutations are more prevalent in never smokers [32]. Smoking is major cause of COPD, and its effect may be attributed to a lower incidence of EGFR mutations; however, we also considered the intrinsic influence of COPD.

In multivariate analysis, the presence of COPD was an independent risk factor for a low prevalence of driver mutations. Furthermore, the analysis of never-smoker patients in our study showed that the prevalence of EGFR mutations was lower in NSCLC patients with COPD, after excluding the effect of smoking. Constant inflammation in the microenvironment and the repair process involved in COPD may be crucial components for the development of cancer related to COPD [33]. Previous studies suggest that inflammation induced by neutrophil elastase or matrix metalloproteinases further contributes to carcinogenesis [34]. Moreover EGFR activation mediates mucus hypersecretion preceded by neutrophil elastase and oxidative stress, thus linking airway inflammation to carcinogenesis [3]. It has been suggested that inflammatory mediators involved in airway chronic inflammation promote malignant transformation of bronchioalveolar stem cells [35,36]. Other possible common pathogenic mechanisms involved in the development of lung cancer in COPD patients include free radical damage of DNA and genetic mutations and polymorphisms [37]. Oxidants that are not balanced by antioxidants lead to DNA damage [38]. Genetic mutation of glutathione S-transferase μ1 (GSTM1), a protective enzyme against tissue damage-inducing substances found in tobacco, was more prevalent in lung cancer patients with COPD than in healthy patients [39]. We suggest that the roles of driver mutations such as EGFR mutations or ALK rearrangements are limited in carcinogenesis in COPD patients.

There were several limitations to our study. First, the percentage of never smokers was disproportionately high in the COPD group, and the predominant overall histological type among the patients enrolled in our study was adenocarcinoma. We routinely test driver mutations associated with non-squamous type NSCLC, and patient data were collected consecutively from the multiple centers. Therefore, we assume that the enrolled patients represent the overall lung cancer patient population. Second, we defined COPD only by spirometric results, such that a disparity may exist between the COPD group in our study and COPD patients on average. However, previous studies regarding lung cancer presenting with COPD also defined COPD by spirometric results alone [30,40,41]. Lung function might be affected by the anatomical location of the lung cancer; however, no significant difference in clinical stage between the COPD group and non-COPD group was detected.

In the present study, lower proportions of EGFR mutations and ALK rearrangements were found in the COPD group compared with the non-COPD group. Moreover, the prevalence of the driver mutations decreased as the severity of airway obstruction increased. The low prevalence of EGFR mutations among COPD patients without a smoking history suggests that NSCLC presenting with COPD may be a separate phenotype of lung cancer. Further studies are required regarding the molecular mechanisms of lung cancer associated with COPD.

## Supporting Information

S1 Dataset Dataset_COPD_drivermutation.xlsx. This is the minimal dataset.
(XLSX)
